# Patients’ perspectives on adherence to cardiovascular screening consultation and lifestyle changes

**DOI:** 10.1186/s13690-024-01256-x

**Published:** 2024-03-06

**Authors:** Julie Katrine Karstensen, Ann Bremander, Katrine Engholm Nielsen, Jette Primdahl, Jeanette Reffstrup Christensen

**Affiliations:** 1 Department of Regional Health Research, University of Southern Denmark, Odense, Denmark; 2 Danish Hospital for Rheumatic Diseases, University Hospital of Southern Denmark, Sønderborg, Denmark; 3 Section of Rheumatology, Department of Clinical Sciences Lund, Lund University, Lund, Sweden; 4 Spenshult Research and Development Centre, Halmstad, Sweden; 5 User Perspectives and Community-based Interventions, Department of Public Health, University of Southern Denmark, Odense, Denmark; 6 Sygehus Sønderjylland, University Hospital of Southern Denmark, Aabenraa, Denmark; 7 Research unit of General Practice, Department of Public Health, University of Southern Denmark, Odense, Denmark; 8 Research Unit for General Practice, Aarhus University, Aarhus, Denmark; 9 DRIVEN - Danish Centre for Motivational and Behaviour Science, Department of Sports Science and Clinical Biomechanics, University of Southern Denmark, Odense, Denmark

**Keywords:** Behaviour change, Cardiovascular risk management, Health services research, Rheumatoid arthritis

## Abstract

**Background:**

Rheumatoid arthritis (RA) poses a significant health burden, with patients facing a twofold higher risk of cardiovascular diseases compared to the general population. As a results, the international recommendations set forth by the European Alliance of Associations for Rheumatology, advocate for a structured cardiovascular (CV) risk management and adherence to a healthy lifestyle for patients with RA. Unhealthy lifestyle factors not only impact overall health but also worsen inflammation and hinder treatment response in patients with RA Despite these recommendations, there remains a knowledge gap regarding patients’ attitudes towards screening participation and lifestyle changes. Therefore, the aims of this study were firstly to explore the perspectives of patients with rheumatoid arthritis on participation and adherence to cardiovascular screening. Secondly, to explore patients’ perspectives on lifestyle changes.

**Methods:**

Semi-structured interviews based on a hermeneutic approach were conducted. The analysis was guided by qualitative content analysis, employing an inductive approach.

**Results:**

Nine women and seven men, aged 47 to 76 years, diagnosed with RA, and who had attended at least one CV screening session, took part in the study. Two primary themes, along with four sub-themes, emerged from the analysis. The first main theme, *Accepting an offer,* encompassed the sub-themes of *Engagement in the screening consultation* and *Risk awareness*, reflecting participants' views on their involvement in, and commitment to, CV screening. The second theme pertained to participants' perspectives on lifestyle changes: *Living with a chronic disease and embracing changes*, described through the sub-themes of *Motivation for lifestyle changes* and *Strategies to achieve lifestyle changes*.

**Conclusion:**

Motivations for taking part in the screening differed among the participants, ranging from simply accepting an invitation to joining as a proactive precaution. In general, there was unanimous agreement among the participants that the screening proved to be a positive encounter. While it may not have immediately prompted significant lifestyle alterations, it did enhance their awareness of risks and underscored the significance of maintaining a healthy lifestyle. Overall, the individual guidance and support for patients with rheumatoid arthritis should entail awareness of CV risk combined with support to lifestyle changes the participants want to pursue.

**Supplementary Information:**

The online version contains supplementary material available at 10.1186/s13690-024-01256-x.

**Table:** 

**Textbox 1. Contributions to the literature**
• Motivation for participating in cardiovascular screening is shaped by individual factors such as available time, a desire to contribute for others, potential benefits, and awareness of risk.
• The adherence to lifestyle changes appears to be connected to biopsychosocial areas: individuals' awareness of their own cardiovascular risk, the presence of social support, and perceived benefits.
• This study underscores the significance of tailoring support for rheumatoid arthritis patients according to their unique values and preferences.
• Promoting cardiovascular risk awareness through visual aids and integrating discussions about cardiovascular risk into conversations on the importance of maintaining a healthy lifestyle is crucial.

## Background

Patients with rheumatoid arthritis (RA) have a doubled risk for cardiovascular diseases (CVD) compared to the general population [1]. Both the inflammatory burden and traditional risk factors contribute to this increased risk [2]. Unhealthy lifestyle factors such as smoking, excessive alcohol consumption, obesity, and low levels of physical activity affect general health but can also affect inflammation and treatment response negatively [2]. International guidelines from the European Alliance of Associations for Rheumatology (EULAR) endorse structured cardiovascular (CV) risk management for patients with RA, including screening for risk factors and to highlight the benefits of a healthy lifestyle [2].

Based on the EULAR recommendations and national guidelines, all patients under the age of 75 are invited for a systematic nurse-led CV screening at the Danish Hospital for Rheumatic Diseases [2–4]. The CV screening consultation aims to inform patients about their increased risk of CVD and discuss their overall risk profile and their motivation for potential lifestyle changes [5]. The risk profile is evaluated using the Systematic Coronary Risk Evaluation (SCORE) [6]. Using the SCORE scheme, nurses calculate and discuss the patient’s risk for CV death based on a graphic chart [6]. The risk score is multiplied by 1.5 in patients with RA for a more accurate risk prediction [2]. A risk SCORE of <5% is considered to represent a low to moderate risk, and a risk SCORE of ≥5% is considered a high to very high risk for CV death within 10 years [2, 6]. Blood tests are performed before the screening, and the nurse explores the patient’s values, priorities, possible support, and barriers to lifestyle changes during the consultation [3, 4]. In agreement with the EULAR recommendations, patients under the age of 70 are invited for a follow-up session based on their calculated risk SCORE at the initial CV screening [2]. Patients with a high risk are invited for follow-up after one year, and patients with a low risk every second or third year, based on the presence of modifiable risk factors [2]. Patients are referred to their general practitioner (GP) if they need pharmacological treatment, or if the patient is at high CV risk. If the patients are interested in additional lifestyle change support, the patients are referred to their GP and are informed about the opportunities for support in their municipality’s health care centres.

Earlier studies indicate that participating in CV screening and adhering to lifestyle interventions can be difficult [7, 8]. Changing lifestyle is a well-known challenge that affects everyday life [9]. In a previous study, we found that one in every two patients with RA reported two or more unhealthy lifestyle factors [10]. According to patients with RA, the greater the number of unhealthy habits, the more challenging it becomes to change their lifestyle [11].

Overall, participation in CV screening and support for subsequent lifestyle changes are essential components of comprehensive care for patients with RA. To improve CV screening and support patients with RA in lifestyle changes to reduce their CV risk, it is crucial to understand their perspectives and motivation for participation. Hence, the aims of this study were first, to explore the perspectives of patients with RA on participation in, and adherence to, CV screening, and second, to explore the patients’ perspectives on lifestyle changes.

## Methods

This study was planned as a qualitative explorative study based on a hermeneutic approach to interpret the data [12, 13]. This approach encompasses the researcher's preunderstandings, the context, and the interpretation of the meaning of the text in a process that moves back and forth between the parts and the whole when interpreting the data [13–15]. The study is reported in accordance with the COREQ 32-item checklist, a comprehensive guide for evaluating and ensuring the transparent reporting of various elements in qualitative research studies [16].

### Setting and recruitment

**Fig. 1:**
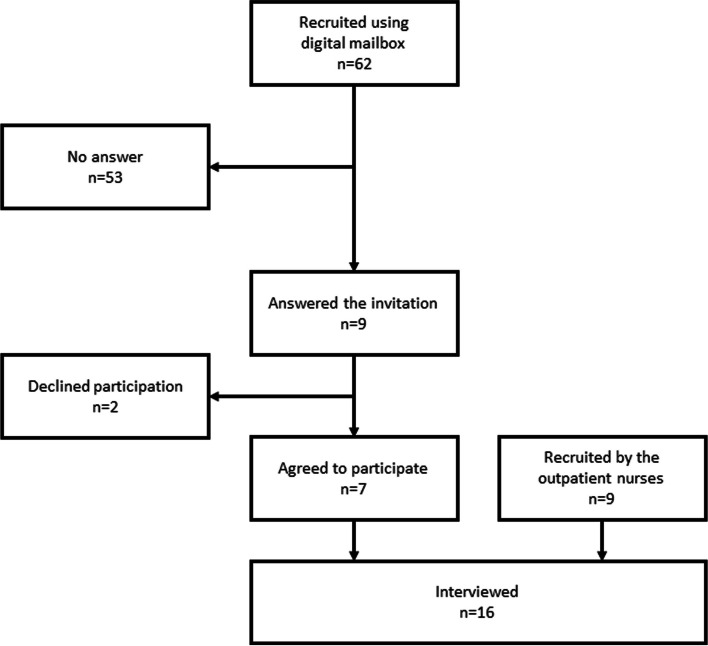
Flowchart of the recruitment of participants

Participants were recruited from the outpatient department at the Danish Hospital for Rheumatic Diseases between January and July 2022. Patients diagnosed with RA by a rheumatologist based on the American College of Rheumatology criteria [17] and who had participated in at least one CV screening consultation between 2019 and 2022 were included in the study. Patients were excluded if they had severe cognitive problems or did not speak Danish. Between July 2012 and July 2015, a total of 917 patients with RA participated in at least one screening consultation at the Danish Hospital for Rheumatic Diseases [7]. Among these patients, 582 were classified as having a low CV risk, while 335 were classified as having a high CV risk according to SCORE [7]. For this study, initially, only patients with high CV risk according to SCORE who were invited to a follow-up CV screening were invited. These patients were identified using the Danish Rheumatology quality database (DANBIO), and an e-mail was sent to eligible patients through the patients’ electronic mailbox (e-box). In total 62, patients were identified via DANBIO, of these 53 patients did not answer the invitation, 2 declined and 7 patients agreed to participate in the study. As the recruitment of patients with high risk proved to be difficult, we chose to include all patients with RA who had participated in a CV screening consultation, regardless of their risk, from March 2022. The second phase of recruitment was done by the outpatient nurses, who informed the patients about the interview study and offered them an information letter immediately after their CV screening. The outpatient nurses recruited further nine eligible patients, please see Fig. 1.

All interested patients, regardless of the recruitment method, contacted a research secretary in the research department of the Danish Hospital for Rheumatic Disease. The secretary offered additional information about the study and informed the interviewer (first author, JK). All interested patients were included in the study without any exclusions. The first author contacted all interested patients by telephone, where additional information was offered, and a declaration of consent was obtained. Figure 1 shows the recruitment process.

### Data collection

After the first author received the signed declaration of consent, a date and place were agreed upon for the interview with each participant. The individual semi-structured interviews could be held at the hospital, via telephone, or online, according to the patients’ preferences. All interviews were conducted approximately one week after the initial contact with the participant. An interview guide was developed in collaboration with our patient research partners, according to the aims of the study and existing literature on the topic [4, 18]. The interview guide consisted of open-ended questions regarding the participants’ experiences of participating in the CV screening and lifestyle changes (see Supplementary File 1). All interviews were conducted by the first and the third author. The participants were encouraged to ask questions and were informed of the option to decline to answer questions if they preferred not to respond. All interviews were audio recorded and transcribed verbatim by the first and third author, in NVivo, using a set of predefined transcription rules. These rules encompassed accurately capturing the spoken words of the patients, employing specific symbols for diverse sounds and pauses, and maintaining a consistent format throughout the transcription process.

### Data saturation

In this study, data saturation was systematically monitored and achieved through a rigorous approach to data collection. Guided by pre-established principles, our emphasis was on delving into the depth and ensuring the quality of the data to achieve a comprehensive understanding of the topic [19]. During the last four interviews, no additional issues were introduced. This observation suggested that the inclusion of additional participants would not have yielded substantially greater insights or information.

### Data analysis

The analysis was inspired by qualitative content analysis as described by Graneheim and Lundman [15]. This systematic analysis aimed to delve into both the explicit (manifest) and underlying (latent) dimensions of the data. Step 1 involved reading all the data material, where interviews, serving as the unit of analysis, were comprehensively reviewed alongside listening to recorded interviews to grasp the entirety of the information. In Step 2, relevant meanings or phrases, along with their contextual surroundings, were identified and extracted as meaning units. These meaning units contained information pertinent to the study's objectives. In Step 3, the meaning units were condensed and assigned specific codes. These codes were then organized into subcategories, further grouped into categories based on similarities and patterns, constituting the manifest content. Finally, in Step 4, these categories were clustered into themes that derived an interpreted meaning [15]. The analytical process was dynamic, involving continuous revisiting and reevaluation of findings as new insights emerged during the analysis. Discussions within the author group contributed to refining interpretations, ensuring that conclusions remained nuanced and accurate over time. The software program, NVivo version 12, from Alfasoft.com, was used to organise and facilitate a structured analysis.

## Results

Sixteen patients with RA were interviewed, nine women and seven men, and all agreed to a follow-up CV screening. The duration of the interviews was not predetermined before the interviews took place. The duration of each interview varied, ranging from 15 to 59 minutes with a median duration in 28 minutes. Due to the COVID-19 pandemic the participants were provided with the flexibility to choose among face-to-face interviews, online, or telephone interviews. Notably, a majority of participants—specifically, nine in total—opted for telephone interviews. Importantly, it is worth highlighting that conducting interviews over the phone did not seem to compromise the quality or depth of information gathered, a perspective supported by Novick [20]. Additionally, one participant chose an online interview, while six participants opted for face-to-face interviews conducted at the hospital. The participants’ sociodemographic characteristics are presented in Table 1.

**Table 1:** Sociodemographic characteristics of the included participants

Characteristics	Number
Age, years, median (range)	71 (47–76)
Gender, Women, *n* (%)	9 (56)
Disease duration, years, median (range)	18 (6–40)
Living with someone, *n* (%)	10 (63)
Employment status, *n* (%)	
Fulltime	4 (25)
Early retirement	3 (19)
Retired	9 (56)
SCORE, *n* (%)	
Low risk: SCORE <5	9 (56)
High risk: SCORE ≥5	7 (44)

*SCORE* Systematic Coronary Risk Evaluation (SCORE multiplied by 1.5 in patients with RA for a more accurate risk prediction) [2]

**Fig. 2:**
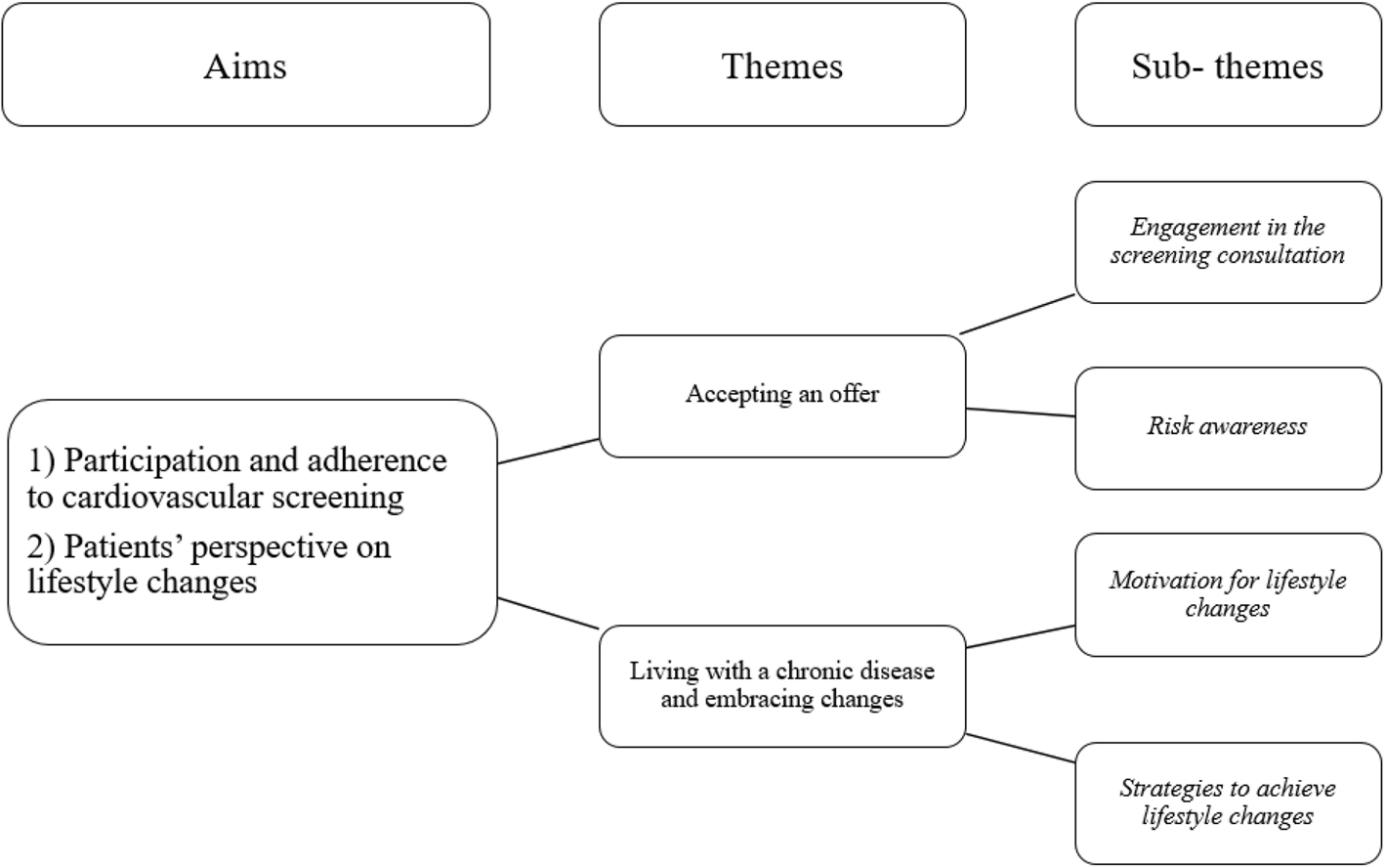
Aims of the study, and the themes and sub-themes derived from the analysis

The analysis derived two main themes: A*ccepting an offer* and L*iving with a chronic disease and embracing changes*. *Accepting an offer* was described by two sub-themes: *Engagement in the screening consultation* and *Risk awareness*. *Living with a chronic disease and embracing changes* was described by the sub-themes M*otivation for lifestyle changes* and S*trategies to achieve lifestyle changes* (Fig. 2).

In the following, the main themes and sub-themes are described in detail and illustrated by selected quotes.

### First main theme: accepting an offer

The participants described different reasons for taking part in the screening. Furthermore, some participants described the awareness and understanding of the CV risk as the reasons for accepting both the initial invitation but also adhere to the follow-up.

### Sub-theme: engagement in the screening consultation

The decision to participate in CV screening was based on factors such as having the time for it, that the screening will do no harm, or simply that it was an offer presented by the hospital.

*It's a screening that is done every 3 years. And it was 3 years ago since I last had it done, and it's just part of the package at the Danish Hospital for Rheumatic Diseases. (Male, high-risk)*

For others, their engagement in the screening was motivated by the desire to positively contribute to the benefit of others with the same diagnosis.

*If one can contribute to something positive for others and perhaps gain something from it oneself, isn't that the hope? That's what I'm expecting to come out of it, at least. (Male, high-risk)*

Furthermore, for some of the participants, the screening served as a means of conducting a status check to ensure everything was in order and determine if any modifications were required. The consultations served as a means for participants to engage in periodic monitoring, acting as a proactive measure to address potential issues before they could escalate into significant problems.

*Well, it's not a pleasant thought to have, but then I think that if I get checked occasionally to see if it has settled anywhere, then I'm aware of it and will react if something is wrong. (Female, low-risk)*

The participants regarded their consultation with the nurse as a good experience and expressed a sense of appreciation towards the nurses. They found the nurses to be pleasant and kind during the interaction, which contributed to their overall satisfaction with the consultation. Some participants felt that the screening did not provide any new insights, as they were already well informed. Some were even concerned that discussions on lifestyle and the measures taken might make individuals feel judged and that it could be intimidating.

*The only thing one could think during this conversation was: "Oh no, now you'll have to be weighed and found too heavy," and that might discourage some people. (Female, low-risk)*

A feeling of being disappointed that they were not invited for yet another follow-up screening was also described. Before inclusion in this study, a few participants had declined further follow-up. They explained that it was because the screening was the same every time.

*Oh, once a year I think... Yeah. But I don't do it anymore. It's over... It was the same we talked about every time. It doesn't matter, I'm doing fine. (Female, low-risk)*

### Sub theme: risk awareness

One of the reasons for accepting an invitation for screening was the awareness of the increased risk of CVD in patients with RA, a family history of CVD, and the desire to gain knowledge about the associated risk factors and preventive measures. This understanding of the risks and the opportunity to learn about prevention served as a primary reason for their participation and willingness to engage in follow-up consultations.

*Yes. I had a certain fear that it had gotten worse. I mean, it wasn't good to begin with, and then I thought, "Hey, maybe I should just check if it had gotten worse, you know." I don't really feel like ending up like a vegetable because of a heart issue, heart failure, you know. So that was the reason why I wanted to see if it had gotten worse. (Male, high-risk)*

The participants recalled their risk for CVD being presented via the SCORE risk chart, which was described as an effective and easily comprehensible method for illustrating their risk by the participants.

*The risk is clearly shown on the chart with the green fields, where everything was fine, and then a red one appeared. So, it was proven on paper, you could say. I thought that was very good. (Male, high-risk)*

When directly questioned about their recollection of the risk SCORE calculated by the nurse, most participants could remember their risk assessment. While some participants faced challenges in recalling the exact numerical value, they were able to recall whether their risk fell into the green, yellow, or red category, or if it was classified as low, moderate, or high. The scheme's emphasis on risk visualization was enhanced by the use of colors, notably observed when the risk level transitioned from green to red upon a participant's disclosure of being a smoker, highlighting the impact of smoking on their risk.

*Yes, everything was green until they mentioned that I smoke. Then a red one appeared. (Female, high-risk)*

The familiarity of these colors, commonly associated with "danger" for red and "good" for green, facilitated the participants' recognition and interpretation, as they are frequently employed in various contexts outside of the screening, making them easily understandable for the patients.

*I don't necessarily need it in color, but, you know, we're familiar with the system from traffic lights with red, yellow, and green. You can say, "Now I'm in the green, that's where I should be." There's a light green and a dark green, where it shifts from 0 to 1, but that's related to age, and people can easily understand that. The darker it gets, the worse it is. (Male, low-risk)*

For some, the scheme was a helpful repetition and provided a comprehensive explanation of the numbers.

*Well, I think that's good because then you can also see the result from last time, and you can still track whether you are on the right track, so to speak. (Female, low-risk)*

For others, the illustration of their risk made them intentionally avoid looking at the scheme. A few participants stated that there was no discussion regarding the risk SCORE during their screening. Other participants admitted that they had not given much thought to the conversation with the nurse. The risk of CVD was perceived as a highly serious matter, but also that risk is just a part of life. However, some participants felt no need for any changes, despite their risk.

*Um, somewhere between quite high and high (laughs). Yeah, well, they claim, persistently, that I was, or am, overweight. And that's it. But, as I said, those cardiovascular diseases haven't really made me lose weight. (Male, high-risk)*

The need for further attention and support for those who were at high risk and to ensure they received appropriate interventions and resources to decrease their CV risk were stated by some of the participants. One participant emphasised that simply receiving good advice and information on CV risk are insufficient to drive lasting changes and felt that knowing that you are accountable to someone or receiving regular check-ins makes it easier to stay on track.

Attending other offers were also a part of the discussion with the nurse and some had been referred to other health professionals i.e., a dietician. Many of the participants were either already visiting their GP or were recommended or informed by the nurse of the opportunity and importance of attending yearly visits with their GP due to their CV risk. Some participants discussed the necessity of attending both the screening and the yearly GP visit, where a yearly check-up with the GP was preferred by some. Others described a feeling of not getting the same type of information on CV risk when attending the screening and at the GP.

*Actually, it has been nice to be able to talk with her [the screening nurse] about it. Especially because you don't have the same conversations with your own GP. You just don't, and not even with the doctor here [rheumatologist at the hospital]. There were two years when I wasn't here at all, and the last two years I've had different doctors. So, it's not easy either. You don't really establish a relationship with them. (Female, low-risk)*

In addition to the screening, some participants had expected and wanted a more extensive check of the heart to be incorporated. Some were left with a fear of not knowing if everything was all right in terms of their heart.

*You don't have the sensation of: "Wow, this [the CV screening] is wonderful. Currently, there's nothing wrong," even though you know the risk is there. I don't know if there's anything wrong. Not even after that conversation. (Female, low-risk)*

### Second main theme: living with a chronic disease and embracing changes

Motivation for lifestyle changes was described in terms of a desire for change, and the need to prioritise making changes in everyday life. The importance of awareness of their CV risk and a social network were mentioned as strategies to achieve lifestyle changes in terms of physical activity and potential adherence to lifestyle changes.

### Sub theme: motivation for lifestyle changes

Quitting smoking, adopting healthier eating habits, and increasing physical exercise were mentioned as areas for improvement where the participants wished to make changes. During the screening sessions, the nurse provided participants with lifestyle recommendations and suggested areas for potential changes. The recommendations emphasised the importance of a healthy diet, with specific instructions on dietary changes and a better understanding of nutrition to manage cholesterol levels effectively.

*They talked about what cholesterol levels and nutrition can do for cardiovascular diseases. (Male, low-risk)*

A key recommendation was to engage in activities that elevate their heart rate and incorporate more exercise into their daily routines. Following the conversation, many participants reported an enhanced focus on physical activity or disclosed that they had already prioritised it, attributing this change to improved emotional well-being and its positive impact on their everyday lives and disease management. The participants recognised the value of physical activity in enhancing their overall well-being, including its potential to improve symptoms related to their diagnosis.

*It's probably the physical activity because I feel that it benefits me. (Female, low-risk)*

They also recalled talking to the nurse regarding alcohol consumption. Several participants stated that they did not have any issues with alcohol and only drank alcohol on special occasions or sporadically. Many of the participants mentioned limited alcohol consumption due to medication and the potential risk of liver damage. Despite being aware of the recommendations, external factors or specific situations could potentially influence their decision-making, causing them to deviate from the recommendations.

*Yes, of course, we did. Alcohol in relation to what I'm dealing with, in connection with the medication I'm taking, it's not a good combination. But every now and then, you forget about that when the party is good. (Male, high-risk)*

The participants acknowledged that changing a lifestyle habit is a difficult task, with one individual going so far as to describe quitting smoking as the most challenging experience he had ever faced. According to the participants, the motivation must originate from within the individual. They expressed that true changes are possible when an individual is genuinely motivated and driven to make changes.

*Yes, the only thing that really needs to happen is for me to pull myself together and get it done. I think it was called character strength in my childhood. (Male, high-risk)*

Making small changes posed no difficulty for some of the participants. Others firmly believed that their current lifestyle did not require any modifications. They felt they were already physically active or felt content with their present lifestyle. Some felt that making changes would make no difference anyway due to old age and medication. In addition, some found it challenging to initiate any changes as they were satisfied with their current lifestyle.

*I think changing lifestyle will be difficult for me. I might as well say it, because I feel good now, and why the hell should I change that? (Male, high-risk)*

### Sub theme: strategies to achieve lifestyle changes

Some participants shared that their awareness of the risks and consequences associated with their unhealthy lifestyle habits, such as smoking or eating too much, served as a motivator to make changes. For some, the fear of relapsing and being on the edge served as a pivotal point to quit smoking. The strategies to quit smoking included adopting new routines and using distraction techniques. The participants acknowledged that they possessed knowledge regarding which lifestyle changes they considered beneficial or detrimental, particularly in relation to CV risk, but it did not always lead the lifestyle changes.

*They've mentioned it, but apparently it hasn't fully sunk in. (Male, high-risk)*

Quitting smoking was described as a difficult challenge. One participant shared the experience of using a bet with another person as motivation to quit smoking.

*We had a bet. I had to work on New Year's Eve, and so did the other person. Ten minutes to twelve, I smoked my last cigarette. (Female, low-risk)*

The participants described that in order to become more active, one must actively engage in activities and make a conscious effort. Embracing an active lifestyle, prioritising exercise, and simply being more physically active were common descriptions among the participants.

*I might consider, every now and then, that even though I'm quite active now, I could incorporate a bit more exercise than I currently do… (Female, low-risk)*

Furthermore, the participants emphasized the significance of being active with others and having a source of motivation when it came to physical activity. The presence of a supportive social network had a profound impact on the participants, with the majority expressing a preference for engaging in physical activity with others. They viewed physical activity as a social endeavor, where companionship played a crucial role. One participant mentioned that living with someone who was highly active had a positive influence on their own activity levels. Participating in physical activity with others was perceived as a commitment, fostering a sense of accountability.

*At least having someone to do it with. It adds a sense of commitment when you have someone… We take turns driving. There are two of us. When you're alone, you might not go [exercising]. (Female, low-risk)*

## Discussion

The reasons for accepting the offer to take part in a screening consultation varied among the participants, from just accepting the offer to participating as a preventive measure. Overall, there was a consensus among the participants that the screening was a pleasant experience, not necessarily leading to lifestyle changes, but that it increased their awareness of risk and the importance of a healthy lifestyle. Some participants saw the screening as a health check or as a proactive measure to address potential issues before they escalated into significant problems. This aligns with the findings from other studies [4, 21], where the potential for benefits leads to participation in screening programs [21]. To contribute for the benefit of others also seemed important for some in this study, as was also seen in a similar study [4]. The recruitment of participants in this study started with only patients at high risk for CVD who had either accepted or declined a follow-up screening. However, this proved difficult, and none of the participants at high risk who declined a follow-up agreed to participate.

There is limited knowledge on non-participation in lifestyle intervention and preventive programs, especially in patients with RA. Previous studies on non-participation in lifestyle related-interventions or prevention programs have found that a reason for non-participation is lack of time [22–25]. This may be due to a busy schedule, where work and the extensiveness of the program may be a barrier [25]. After participating in this study, a few participants declined further follow-up. Their reason was that the screening was the same every time or that they attended yearly follow-ups with their GP instead. In a Dutch study, only 16% of patients with RA and high risk contacted their GP after a CV screening [8]. However, a previous study from the Danish Hospital for Rheumatic Diseases found that having RA increased the odds of contacting their GP, with 76% of high-risk patients doing so after a CV screening [7]. Of those who contacted their GP, 38% received consultations that led to relevant actions concerning their CV risk [7]. This was only related to blood samples, blood pressure, etc., and it is not known if the GP also talked to the patients regarding lifestyle changes. A Swedish study from 2018 found that registered nurses are more likely to use clinical practice guidelines to support lifestyle management than GPs are [26]. Notably, the nurses who carried out the CV screening consultations in this study are trained rheumatology nurses who place special emphasis on identifying patients' values, preferences, resources, motivation and opportunities for implementing lifestyle changes in their daily lives. Consistent with this approach, other studies have underscored the importance of tailoring interventions to the individual [27, 28]. Some participants in our study expressed that they did not get the same extensive follow-up with their GPs, highlighting the need for individualised offers and explanations, such as in the CV screening program at the Danish Hospital for Rheumatic Disease, to address the increased CVD risk among these patients and discuss motivation for changes in lifestyle.

Some participants left the screening with a fear that something was wrong with their heart, and a more extensive heart check was suggested as part of the screening offer in the future. Another study also found that patients accepting similar screening offers did not feel that they had been examined properly [4]. This indicated that the patients expected a different outcome from the screening than what they experienced [4]. Illustrating this, the patients in this study understood that the results from the screening, including that the risk SCORE only expresses an estimation, not a definite result of their actual risk. However, the SCORE risk chart, with its illustrations and colour coding, seemed to enhance participants’ comprehension of the risk and potential risk factors. The familiarity of the colours helped the participants to recognise and interpret the information given, as they are frequently employed in various contexts. A similar use of the colours red, yellow, and green - traffic light colours - has also been used in other studies, and participants have responded positively, perceiving it as an effective way to communicate risk [29–31]. Mixed feelings were found regarding the chart. It was either seen as a way to keep track, or not wanting to look at the numbers ever again. The negative feeling may have something to do with a high risk for CVD and not wishing to implement lifestyle changes.

The participants described varying degrees of awareness regarding healthy living after attending the screening. While some participants felt they already had knowledge of a healthy lifestyle, others increased their awareness of the importance of a healthy diet and increased physical activity. In other studies, awareness of physical activity has also been identified as a motivating factor for patients with RA [32, 33]. A European-based survey found that the most important facilitators of physical activity among people with rheumatic and musculoskeletal diseases were less fatigue and pain, and the ability to perform activities more easily [34]. Similar statements were found in this study, where the participants described improvement in their overall well-being following an increase in physical activity. A previous study explored changes between the first and second screening in patients with inflammatory arthritis [35]. The study found that 18% of the patients at high risk and 20% of the patients at low risk increased their physical activity from <5 to ≥5 times a week [35]. The study also explored changes in smoking habits and found that 20% of high-risk patients and 15% of low-risk patients, who had reported being smokers at the initial screening, had transitioned to non-smokers by the second screening [35]. These results indicate that the increased awareness and knowledge of the consequences of the habits, as stated by the participants in this study, can contribute to positive changes in lifestyle. Although many of the participants were aware of, and followed, the recommendations for alcohol consumption, some of the participants described consuming alcohol on social occasions knowing the potential consequences. To further complicate the issue of alcohol consumption in patients with RA, recent studies have reported that alcohol consumption can be associated with lower disease activity and higher health-related quality of life [36, 37]. However, limited alcohol consumption is recommended as it can interact with some medications [38].

The participants emphasised the proactive nature of motivation, highlighting that just desiring higher activity levels is insufficient and underlining the importance of taking concrete measures and incorporating physical activity into daily routines to become more active. The self-determination theory developed by psychologists Edward L. Deci and Richard M. Ryan distinguishes between intrinsic and extrinsic motivation [39]. Intrinsic motivation is when an activity is performed because the activity is meaningful for the individual or gives a sense of satisfaction [39]. Extrinsic motivation is when the individual performs an activity or a task because someone else requires it or because one wants to achieve something else than the joy of the specific activity [39]. For the participants in this study, the extrinsic motivation may be related to their CV risk, their calculated SCORE and the recommendations from the nurse. However, some participants in this study felt no need for change. This indicates that some of the participants had no intrinsic motivation for change, as they are satisfied with their current lifestyle and quality of life. A systematic review has found that patients with RA lack awareness of the connection between CVD and RA and that their increased risk for CVD stems from their RA diagnosis [40]. Furthermore, it has been found that patients with RA may underestimate their CV risk [41], which highlights the importance of continuing to promote CV risk awareness in the screening consultations. Furthermore, the social network, especially in terms of physical activity, also influenced the participants’ motivation for lifestyle changes, indicating that psychosocial issues are of great importance for lifestyle and motivation for change, not only the biomedical arguments. Social support as a facilitator for increased physical activity in patients with RA was also found in a literature review from 2015 [42]. Motivation for lifestyle changes seemed to be influenced by external factors such as support and resources, but ultimately, both intrinsic and extrinsic motivation are crucial for sustaining the commitment and perseverance required for changing lifestyle habits. Furthermore, in a previous study we found that one in every two patients with RA have two or more unhealthy lifestyle factors [10]. This highlights the challenge of initiating change, as it necessitates addressing not just one but potentially multiple aspects of their lifestyle. Consequently, it becomes crucial to assist RA patients in identifying their priorities, thereby facilitating their efforts to implement feasible changes in their daily routines.

### Strengths and limitations

Following ethical research principles, participation was entirely voluntary, with inclusion limited to statements from willing participants who have the time and a genuine desire to be involved. This approach prioritizes individual autonomy and informed consent, thereby upholding the overall ethical integrity in the research process. Nevertheless, preventive programs, such as the CV screening at the Danish Hospital for Rheumatic Diseases, often encounter challenges such as limited participation rates and an overrepresentation of women and individuals with better health and socioeconomic status [43]. One of the strength in this study is that in accordance with findings from previous studies, the participants in this study are representative of the patients with RA accepting an invitation to a screening [3, 4]. However, compared to these previous studies, a larger proportion of men agreed to participate in this study. More than half of the participants in this study were retired, which may influence their reason for participating in terms of allocating the necessary time. A limitation of this study may arise from the varying durations between participants' engagement in CV screenings and their subsequent participation in interviews. Specifically, nine participants were interviewed shortly after their CV screening, while seven had undergone screening within the past three years. Although there is the potential for recall bias, impacting participants’ memory of specific details, the interviews provided valuable insights into their perspectives and the lasting impact of CV screening, regardless of the time that had passed since their last screening.

## Conclusion

In conclusion, the reason to participate in a CV screening program varied from just accepting an invitation to a feeling of taking preventive measures for the future. The reasons for attending CV screening were also based on individual factors, such as having the time for it, contributing for others, awareness of risk, or potential benefits. In terms of lifestyle changes, some participants became more aware of the importance of a healthy lifestyle, such as a healthy diet and increased physical activity, after taking part in the screening consultation. However, the implementation of lifestyle changes varied between the participants and seemed to relate to the individual’s awareness of their CV risk, social support and possible benefits. Overall, health care professionals should tailor guidance and support for patients with RA to their values and preferences. This entails promoting CV risk awareness using illustrations, such as the SCORE scheme, and include awareness of CV risk when discussing the reasons for adhering to a healthy lifestyle.

### Supplementary Information

**Supplementary Material 1.** 

## Data Availability

The information substantiating the findings presented in the manuscript is unavailable to the public due to Danish law and ethical considerations. The study’s ethical clearance mandates that the transcribed interviews remain secured in locked files, accessible solely by the research team.

## Abbreviations

COREQ: Consolidated criteria for reporting qualitative research
CV: Cardiovascular
CVD: Cardiovascular diseases
DANBIO: Danish Rheumatology quality database
EULAR: European Alliance of Associations for Rheumatology
GP: General practitioner
RA: Rheumatoid arthritis
SCORE: Systematic Coronary Risk Evaluation
