# Supplementary material for: Patients’ perspectives on adherence to cardiovascular screening consultation and lifestyle changes
Source: Arch Public Health. 2024 Mar 6;82:30. doi: 10.1186/s13690-024-01256-x (PMC10919003; doi:10.1186/s13690-024-01256-x)
Supplement: Supplementary file 1 — Supplementary Material 1. [file 13690_2024_1256_MOESM1_ESM.docx]

| **Interview guide** | |
| --- | --- |
| Introduction: Thank you for participating. The interview is anonymous, and you can choose not to answer questions at any point.  The purpose of this interview is to gain insight into your experience with the cardiovascular screening consultation. Additionally, I would like to discuss your lifestyle habits. I anticipate that the interview will last approximately 45 minutes.  I would like to record the interview in order to transcribe it and analyse it alongside other interviews. Is this okay with you? Do you have any questions before we begin? | |
| **Theme** | **Questions** |
| Background | For how many years have you been diagnosed with rheumatoid arthritis?  Do you work?   - As what? - Number of hours per week?   Do you live alone or with others?   - Who? |
| Participation in screening consultations | Why did you decide to engage in a discussion about the risk of cardiovascular disease?  What were your anticipated outcomes from the screening consultation?  What was your overall experience like during the screening consultation?  Did the screening consultation align with your initial expectations?  What topics were you expecting to address?  What factors motivated your interest in participating in a follow-up discussion about your cardiovascular disease risk? |
| Everyday life | What topics did you cover during the screening consultation concerning your daily life?  What holds significance for you in your everyday life?  How does your daily routine correspond with your leisure, social activities, and work?  What would you imagine as your ideal daily life? |
| Lifestyle habits | What information were you given during the screening consultation about your cardiovascular disease risk, particularly regarding your lifestyle habits?  Did the nurse share information about potential resources or options available to address your cardiovascular disease risk?  - Offers in the municipality, assistance from your doctor, etc.?  If you discussed the possibility of modifying your habits, what are your reflections on your current habits related to smoking, diet, alcohol consumption, and physical activity?  Are you content with your present lifestyle? |
| Making changes | Have you implemented any changes following the screening consultation?   - If so, what motivated these changes, or why did you choose not to make any alterations? - Can you share your successes and challenges in this regard? - What factors influence your ability to modify your habits?   Are there specific habits you are keen on changing?  In your opinion, what's essential for you to transform your lifestyle?  What obstacles and opportunities do you encounter in your daily life when it comes to habit modification?   - Is it feasible, and if so, how could it be achieved?   What serves as motivation for you to adhere to these lifestyle changes?  What type of support do you feel would aid you in altering your habits?   - What kind of support, from whom, do you believe would be helpful, and in what way? |
| Ending | Finally, at the end of this interview, I would like to ask if you remember the risk score you received during the screening consultation about cardiovascular disease risk.  Do you have any questions or comments about the interview?  Thank you for participating in the interview. |
